# Extraction, Characterization, and Antioxidant Activity of Pectin from Lemon Peels

**DOI:** 10.3390/molecules29163878

**Published:** 2024-08-16

**Authors:** Anathi Dambuza, Pamela Rungqu, Adebola Omowunmi Oyedeji, Gugulethu M. Miya, Simon K. Kuria, Sunday Yiseyon Hosu, Opeoluwa Oyehan Oyedeji

**Affiliations:** 1 Department of Chemistry, Faculty of Science and Agriculture, University of Fort Hare, P/Bag X1314, Alice 5700, South Africa; prungqu@gmail.com; 2 Department of Chemical and Physical Sciences, Faculty of Natural Sciences, Walter Sisulu University, P/Bag X1, Mthatha 5117, South Africa; aoyedeji@wsu.ac.za (A.O.O.); gmiya@wsu.ac.za (G.M.M.); 3 Department of Biological and Environmental Sciences, Walter Sisulu University, P/Bag X1, Mthatha 5117, South Africa; kkuria@wsu.ac.za; 4 Department of Business Management and Economics, Faculty of Economics and Financial Sciences, Walter Sisulu University, P/Bag X1, Mthatha 5117, South Africa; yhosu@wsu.ac.za

**Keywords:** pectin, antioxidants, degree of esterification, physicochemical properties, biopolymer, equivalent weight, anhydrouronic acid

## Abstract

Pectin is a natural polymer that is found in the cell walls of higher plants. This study presents a comprehensive analysis of pectin extracted from lemon in two different geographic regions (Peddie and Fort Beaufort) in two consecutive years (2023 and 2024) named PP 2023, PP 2024, FBP 2023, and FBP 2024. The dried lemon peels were ground into a powder, sifted to obtain particles of 500 μm, and then subjected to pectin extraction using a conventional method involving mixing lemon peel powder with distilled water, adjusting the pH level to 2.0 with HCl, heating the mixture at 70 °C for 45 min, filtering the acidic extract, and precipitating pectin with ethanol. The yield of these pectin samples was statistically significant, as FBP 2024 had a maximum yield of 12.2 ± 0.02%, PP 2024 had a maximum yield of 13.0 ± 0.02%, FBP 2023 had a maximum yield of 12.2 ± 0.03%, and PP 2023 had a maximum yield of 13.1 ± 0.03%, The variation in yield could be due to the differences in the growing conditions, such as the climate and soil, which could have affected the pectin content in the lemons. The physicochemical characterization of all samples proved that our pectin samples could be used in the pharmaceutical and food industries, with anhydrouronic acid content which was greater than 65%, as suggested by the FAO. The scanning electron microscope analysis of all extracted pectin was rough and jagged, while the commercial pectin displayed a smooth surface morphology with a consistent size. FTIR confirmed the functional groups which were present in our samples. Thermogravimetric analysis was employed to investigate the thermal behavior of the extracted pectin in comparison with commercial pectin. It was found that the extracted pectin had three-step degradation while the commercial pectin had four-step degradation. Additionally, pectin samples have been shown to have antioxidants, as the IC_50_ of PP 2024, PP 2023, FBP 2023, FBP 2024, and Commercial P was 1062.5 ± 20.0, 1201.3 ± 22.0, 1304.6 ± 19.0, 1382.6 ± 29.9, and 1019.4 ± 17.1 mg/L, respectively. These findings indicate that lemon pectin has promising characteristics as a biopolymer for use in biomedical applications.

## 1. Introduction

Lemons belong to the Rutaceae family, which includes approximately 160 genera of citrus fruits grown across all continents. Globally, citrus production amounts to roughly 85 million tons annually [1]. South Africa is known to be the second largest exporter of citrus fruits after Spain [2]. Citrus fruits hold considerable economic importance in the fresh produce market and as a key ingredient in food processing, with 75% of the total production being utilized for fresh consumption [3]. Fruit processing gives relatively vast quantities of citrus by-products (peels, seeds, pomace, and wastewater), estimated to be between 55% and 60% of the weight of the raw fruit, which has been identified as highly organic and readily susceptible to biological degradation or transformation [3,4]. Aside from that, improper handling, direct disposal, and other strange characteristics of citrus by-products are noted to menace and have an adverse impact on the environment and human health overall [5]. Citrus contains secondary metabolites designed to be a part of their defense mechanism against viruses, fungi, and bacteria [6]. It has long been established that citrus by-products are enriched with biologically active compounds (i.e., vitamins, dietary fibers, pectin, polyphenols, and essential oils). Similarly, these compounds are widely reported to have numerous special applications in industries, such as food, health benefits, pharmaceuticals, cosmetics, and textiles. Several scientific studies reported many pharmacological effects of citrus compounds, such as antioxidant, antimicrobial, anti-inflammatory, anticancer, and antidiabetic properties [7,8,9].

A citrus peel contains pectin, a natural gelling agent commonly utilized in jams, jellies, dairy products, desserts, soft drinks, frozen foods, and pharmaceuticals [10]. Pectin, a naturally occurring polymer in higher plants, can be classified into three main groups based on distinct characteristics [11]. The first group, homogalacturonan (HG), is known as the “smooth region” due to its linear structure consisting of α-(1,4)-linked galacturonic acid. The second group, rhamnogalacturonan-I (RG-I), referred to as the “hair region”, is primarily branched and accounts for 20–30% of the pectin domain. The third group, RG-II, makes up approximately 10% of the total pectin domain and is the most complex group, containing 12 different monosaccharides and over 20 types of linkages. RG-II is characterized by substituted galacturonans (GS) and is considered the most intricate segment of pectin [12,13]. Pectin can be classified based on its degree of esterification, which refers to the ratio of esterified carboxylic acid units to the total carboxylic acid units in the pectin structure [14]. There are two main types, namely low-methoxyl (LM) pectin, with fewer than 50% esterified carboxyl groups, and high-methoxyl (HM) pectin, with more than 50% esterified carboxyl groups. The degree of esterification is crucial as it directly impacts pectin’s structure and functional properties [15]. Pectin was discovered 200 years ago, but determining the exact structure of pectin is quite challenging [16]. Several factors, such as the mode of extraction, source of pectin, pH level, the solvent used during extraction, and the material’s particle size and temperature, significantly impact the structure, quality, and quantity of pectin [17]. Since pectin’s quality and quantity depend upon these factors, many previous studies have compared various techniques for extracting pectin and optimizing it [18,19]. This natural polymer has also been found to possess biological activities. A study by Hu et al. [20] suggested that pectin offers direct protection to β cells by reducing oxidative and nitrosative stress, potentially through inhibiting the pro-apoptotic protein Gal-3 and preventing intracellular danger signal delivery. Their findings indicate that low-methoxyl pectin at a high concentration is particularly effective in safeguarding against oxidative and inflammatory harm, highlighting its potential as an anti-inflammatory agent for pharmaceutical and therapeutic applications. Another study by Brouns et al. [21] demonstrated that citrus pectin reduced insulin resistance in diabetic rats, potentially enhancing insulin sensitivity by regulating key proteins in the PI3K/Akt signaling pathway. This indicates a potential antidiabetic mechanism for citrus pectin. Pectin has been found to possess anti-hypertensive activity [22,23]. Pectin and other soluble dietary fibers (SDFs) impact the glycemic response by slowing down the absorption of glucose in the intestines, leading to a decrease in insulin production by the pancreas. This reduction in insulin levels subsequently decreases the activity of HMG-CoA, resulting in a decrease in cholesterol, which means it could be used as a key ingredient in pharmaceuticals as an anti-hypercholesterolemic agent [24]. Pectin also exhibits anti-Alzheimer’s disease, hepatoprotective, and nephroprotective properties [25,26,27]. However, this current study takes a more comprehensive approach by comparing the effect of seasons on pectin and the impact of the geographic region using conventional extraction from pectin extracted from Peddie and Fort Beaufort in South Africa and commercial pectin in terms of their physicochemical properties and biological activities. Our study integrates multiple analytical techniques (thermal analysis, FTIR, SEM, and physicochemical property examination) to provide a holistic understanding of how geographic and seasonal variations influence pectin’s characteristics. This multifaceted approach is relatively rare in pectin research, which often focuses on one or two aspects.

## 2. Results and Discussion

### 2.1. Pectin Yield

The Peddie pectin 2024 (PP 2024) sample showed the highest pectin yield, reaching 13.0%, while the pectin yield from Fort Beaufort in 2024 (FBP) was slightly lower at 12.2% when compared with PP 2024. The maximum yields of all samples were statistically significant.

In terms of year-to-year variation, the pectin yield from Peddie in 2023 was comparable to that of Peddie in 2024, with both yielding approximately 13.1%. This demonstrates that the Peddie farm has produced constant pectin yields over the last two years. On the other hand, the pectin yield from Fort Beaufort in 2023 was slightly lower at 12.2% compared with Peddie’s yields in 2023 and 2024. The pectin yields obtained in this study are consistent with the recent report of Alencar et al.’s findings. In [28], the authors reported a pectin yield of 12.2%, which was achieved by using the traditional hot acid extraction method with *Spondias tuberosa* L.

The data indicate that the Peddie farm may possess favorable conditions for pectin extraction, evidenced by its consistently higher yields compared with Fort Beaufort’s. The year-to-year consistency of the pectin yields at each farm could be attributed to factors such as agricultural practices, lemon varieties, soil quality, or stable climate conditions over time. Table 1 below demonstrates and summarizes the pectin yields.

### 2.2. Degree of Esterification

Another parameter which has a notable impact on the quality and applications of pectin is the degree of esterification (DE). As shown in Table 2, PP 2023 had a slightly higher degree of esterification (87.0%) compared with PP 2024 (82.7%), while FBP 2023 had a higher DE (89.5%) than FBP 2024 (80.6%), with commercial pectin having the highest DE at 95.5%, indicating a significantly lower degree of methoxylation compared with the farm-specific pectin samples. The significantly higher DE of the commercial pectin compared with the farm-specific pectin samples may indicate a different source or extraction method, leading to distinct pectin properties and applications [29]. All pectin had high DE values. Pectin with a high DE tends to have a lower degree of methoxylation, which results in more free carboxyl groups available for interactions [29]. High-DE pectin has health benefits like supporting digestive health and acting as a prebiotic for beneficial gut bacteria [30]. Commercial pectin (DE: 95.5%) would be most preferred for gelling capabilities due to its high DE, followed by FBP 2023 (DE: 89.5%). DE affects the gelling behavior of pectin, and thus these samples are best suited for applications which require strong gels.

### 2.3. Equivalent Weight

Equivalent weight (EW) means the average molecular weight of the repeating unit in pectin, where a lower EW signifies a greater degree of polymerization, indicating longer pectin chains. PP 2024 had a slightly lower EW value (833.3 g/mol) compared with FBP 2024 (862.1 g/mol), and this EW aligns with the findings of Devi et al. [31], who reported an EW of 833.33 for pectin extracted from sweet lemon peel powder using nitric acid. PP 2023 EW (841.8 g/mol) fell between the EW values of PP 2024 (833.3 g/mol) and FBP 2024 (862.1 g/mol), with FBP 2023 having the highest EW value (983.3 g/mol) and commercial pectin having the lowest EW value (824.2 g/mol), indicating variations in molecular weight distribution within the samples and suggesting shorter pectin chains compared with the other samples. The EW is the total content of free galacturonic acid (not esterified) present in the molecular chains of pectin. The presence of free galacturonic acid in the molecular chains of pectin helps the water-binding properties of pectin [32,33]. In this case, FBP 2023 had the highest EW, signifying more emulsifying properties than the other samples. The reason commercial pectin had a significantly lower EW value was probably the extraction technique employed, as was also evidenced by Wai et al. [34]. Different extraction methods (e.g., hot water extraction, acid extraction, and microwave-assisted extraction) operate under different temperatures and durations. Higher temperatures and longer extraction times can lead to the degradation of pectin (depolymerization and demethoxylation), resulting in a lower molecular weight and, consequently, a lower equivalent weight [35]. Table 2 summarizes the results.

### 2.4. Methoxyl Content (MeO)

As pectin is classified into high- and low-methoxyl types, with their gelling ability varying under different conditions, the methoxyl content (MC) is another key parameter which defines the functionality of the extracted pectin. In terms of the methoxyl content, FBP 2024 exhibited the highest MeO content (19.2%), while PP 2024, as shown in Table 2, had a significantly lower MeO content (9.3%) compared with FBP 2024, PP 2023 showed a moderate MeO content (11.8%), FBP 2023 had a slightly lower MeO content (10.9%), and the commercial pectin had a moderate MeO content (13.7%), with a consistent methoxyl content within each sample. All MeO contents were less than 20%. The most recent study which had an MeO content more than 20% was [36], where citrus peels with a maximum MeO content of 38.8% were extracted. However, most studies have reported citrus peels to possess MeO contents ranging from 5% to 12% [32,36,37,38]. A high methoxyl group content improves the dispersion quality and sugar-binding capacity of pectin [39]. The capacity of pectin to interact with sugars is enhanced by its methoxyl (MeO) content. Higher levels of MeO facilitate more effective interactions with sugar molecules, resulting in increased viscosity and enhanced stability for the final formulation. This means FBP 2024 had the best water binding capacity.

### 2.5. Total Anhydrouronic Acid (AUA) Content

The AUA indicates the purity of the extracted pectin, which should have a value of at least 65% [40]. FBP 2024 had a high AUA content of 94.2% with a consistent distribution, PP 2024 had a slightly lower AUA content of 91.5% with more variability, PP 2023 showed a moderate AUA content of 88.7% with slight variability, FBP 2023 had a lower AUA content of 82.4%, and the commercial pectin had the highest AUA content at 98.9% with remarkable consistency, as shown in Table 2. This means that all of these samples were pure since they had an AUA content of more than 65%, Setting a threshold for the AUA content serves as a quality control measure. Low AUA values suggest that the extracted pectin might have a high amount of protein [41].

### 2.6. Ash Content

FBP 2024 and FBP 2023 had a low ash content of 1.0% with consistent distribution, PP 2024 had a slightly higher ash content of 1.1%, PP 2023 had a similar ash content to PP 2024 at 1.1% with consistent distribution, and the commercial pectin had the highest Aash content at 3.6% with consistent distribution, indicating a significantly higher mineral content. The ash content values indicate the mineral content in the pectin samples, with higher values signifying a greater mineral presence, which can impact the pectin’s quality and properties. The standard deviations show the variability in the ash content within each sample, where lower standard deviations denote greater uniformity in the mineral content. These results are not significantly different from the findings of Konrade et al. [42], with their pectin ash content ranging from 1.94% to 2.65%. The results are shown in Table 2.

### 2.7. Moisture Content

FBP 2024 had a moisture content of 10.8%, PP 2024 had a slightly higher content of 11.1%, PP 2023 had a lower content of 9.6%, and FBP 2023 had a content of 9.3%, which is lower than that of PP 2023 but slightly higher than that of the commercial pectin, which had a moisture content of 9.3%. This variation in moisture content, shown in Table 2, could affect the quality, stability, and storage needs of the pectin samples.

### 2.8. Fourier Transform Infrared Spectroscopy (FTIR) Analysis

**Figure 1:**
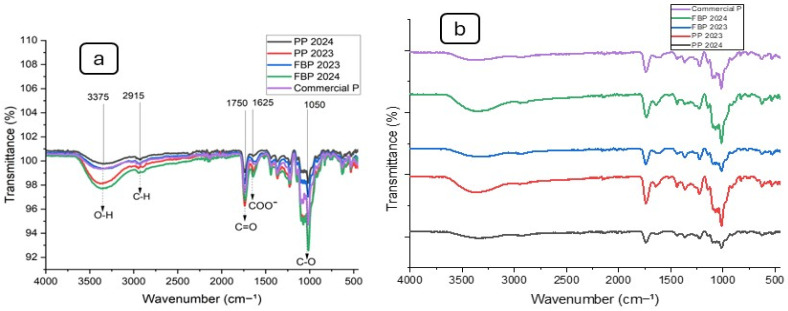
FTIR spectra of pectin samples: (**a**) clustered and (**b**) dispersed spectra.

FTIR was employed to identify the functional groups of the pectin. Figure 1 illustrates that FBP 2024, PP 2024, PP 2023, FBP 2023, and Commercial P all exhibited the characteristic absorption peak of polysaccharides. The FTIR spectra profiles of these five samples were nearly identical. Significant absorption near 3375 cm^−1^ was attributed to stretching of the hydroxyl groups. The absorption at 2915 cm^−1^ was attributed to C–H stretching of the CH_2_ groups from the methyl and methylene groups of polysaccharides. Absorption at 1750 cm^−1^ was due to C=O stretching vibration of the methyl-esterified carboxyl groups. Absorption at 1625 cm^−1^ was caused by C=O stretching vibration of the ionic carboxyl groups in the pectin, and lastly, the C-O stretch at 1050 cm^−1^ corresponded to β-glycosidic linkages [43].

### 2.9. SEM Analysis

**Figure 2:**
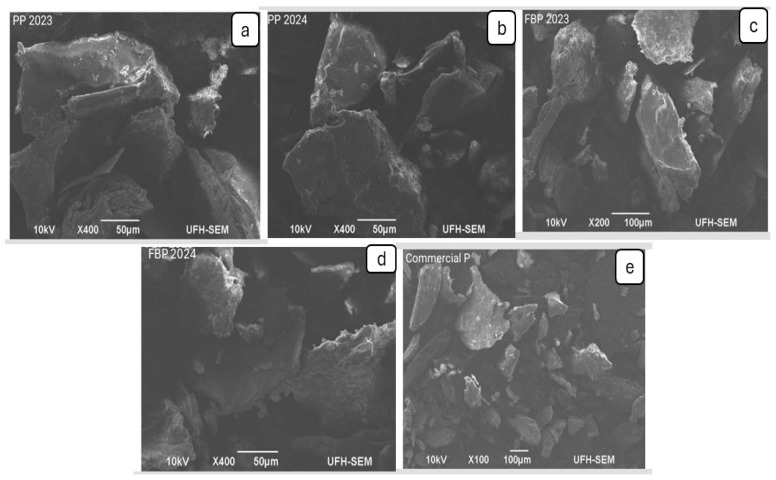
SEM images of pectin extracted from citrus lemon from (**a**) PP 2023, (**b**) PP 2024, (**c**) FBP 2023, (**d**) FBP 2024, and (**e**) Commercial P.

Figure 2 shows the SEM images of four extracted pectin samples: PP 2023 (a), PP 2024 (b), FBP 2023 (c), and FBP 2024 (d), as well as commercial pectin (e). In the SEM images of the pectin particles, size differences significantly influenced the structural characteristics and potential applications of the pectin. The small pectin particles of the commercial pectin displayed a smooth surface morphology with a consistent size and shape. These smaller particles are likely to disperse more readily in solutions, resulting in improved solubility and interactions with other food components. Also, this feature indicates a high surface area, which is beneficial for rapid hydration or binding applications [43,44].

On the other hand, all four extracted pectin samples showed the same morphological features; the SEM images of the four extracted pectin samples were all large, rough, and irregular. Citrus fibers with a large surface area can accommodate more water molecules through hydrogen bonds or dipole formation [45].

### 2.10. Thermal Properties

**Figure 3:**
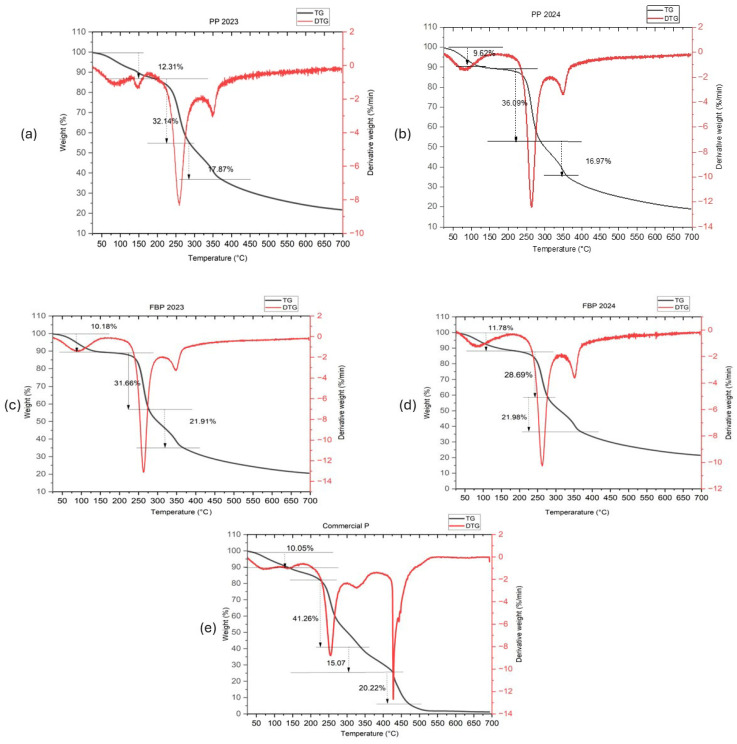
TGA curves of extracted pectin (**a**–**d**) and commercial pectin (**e**).

The thermal decomposition behavior of various pectin samples was analyzed through TGA. The thermal stability of pectin is crucial for its application in drug delivery. TGA analysis revealed the temperature ranges where the pectin maintained stability, ensuring its safe use in pharmaceutical formulations, as shown in Figure 3. The results showed distinct stages of decomposition for each sample. All four extracted pectin samples (PP 2023, PP 2024, FBP 2023, and FBP 2024) had three-step degradation. Initially, weight loss was observed at approximately 80–90 °C, indicating the evaporation of moisture or volatile components [46]. This was followed by significant weight loss at temperatures ranging from 250 to 350 °C, which could be attributed to the breakdown of pectin molecules or the release of organic compounds [47,48]. Finally, the last decomposition stage occurred at temperatures above 350 °C [49]. Table 3 summarizes these three-step degradation processes with their corresponding temperatures. However, commercial pectin had four-step degradation process. At 80.37 °C, the initial decomposition of Commercial P resulted in 10.05% weight loss, corresponding to the evaporation of moisture or volatile impurities. At 254.15 °C, the second decomposition stage was linked to the breakdown of pectin molecules or the release of organic compounds, resulting in a significant loss of 41.26%. At 328.43 °C, the third decomposition stage indicated further degradation of organic matter, with weight loss of 15.07%. At 427.79 °C, the final decomposition stage led to a 20.22% weight loss, suggesting complete degradation of the remaining organic components in the pectin sample.

### 2.11. DPPH Antioxidant Evaluation

The significance of pectin’s antioxidant properties in biomedical applications led us to assess its antioxidant activity using the DPPH method. The compound 2.2-diphenyl-1-picrylhydrazyl (DPPH) is a stable free radical molecule with delocalized electrons, giving its solution a purple color. Upon interaction with antioxidant compounds, DPPH is reduced, turning the solution yellow as DPPH-H is formed [50]. In this process, DPPH serves as a hydrogen donor for the antioxidant [51]. It was found that pectin has some antioxidant properties, but the IC_50_ values of all pectin samples (as shown in Table 4), including the commercial pectin, were less than the IC_50_ of ascorbic acid (15.2 ± 0.3 mg/L), which is a standard antioxidant.

## 3. Materials and Methods

### 3.1. Raw Materials

Citrus lemons were harvested from the Fort Beaufort and Peddie farms (South Africa), with GPS coordinates of 32°45′58.7988″ S and 33°2′11.34.5516″ S, respectively, in July 2023 (winter), with a subsequent collection period in May 2024 (autumn) from the same farms directly after fruit picking from the lemon trees. The lemon fruit powder preparation involved peeling and washing the fruits to remove dirt, extracting the albedo (white part of the peel), blanching the albedo in boiling water for 5 min to deactivate its enzymes, filtering the pieces through two layers of muslin cloths to remove insoluble materials, pressing the pieces by hand to eliminate excess water, drying the resulting alcohol-insoluble solids (AIS) from the lemon peel at 60 °C in a tray drier until a constant weight was achieved, grinding them into powder, and storing them in a tightly sealed, airtight zipped plastic container at room temperature until they were utilized. All chemicals, including the commercial pectin, were obtained from Sigma-Aldrich (Pty) Ltd. (St. Louis, MI, USA) and Merck (Pty) Ltd. (Darmstadt, Germany) in Johannesburg, South Africa through an authorized local distributor: Shalom Laboratories and Supplies in Johannesburg, South Africa.

### 3.2. Extraction of Pectin

The extraction of pectin was achieved using the method of Georgiev-Assen et al. [52] with modifications. Briefly, the lemon fruits were initially peeled and washed to eliminate any contaminants. The peel (albedo) was then subjected to a blanching process using boiling water for 5 min to deactivate its enzymes. Subsequently, the peels were filtered manually through two layers of muslin cloth to eliminate insoluble materials. The resulting alcohol-insoluble solids (AIS) obtained from the lemon peel were dried at 60 °C in a tray drier until a consistent weight was achieved. The dried solids were then pulverized into a powder using a grinder, and sieves were utilized to collect lemon powder particles 500 μm in size. Then, 5 g of lemon peel powder was mixed with 150 mL of distilled water in a 250 mL conical flask, and the sample was then shaken for 1 min. The pH was adjusted to 2.0 using 2 M of HCl, and the mixture was heated in a water bath at 70 °C for 45 min. The resulting acidic extract was filtered through a cloth, and the pectin-containing aqueous extract was precipitated by adding an equal volume of 99.1% ethanol. Then, the sample was left at 4 °C for 3 h. The solid material was collected by filtration and washed with a 1:1 volume of 75% ethanol.

The pectin yield was calculated using the equation below. Tests were performed in triplicate, and the average yield was used:

$$\mathrm{Pectin}\;\mathrm{yield}\;(\%)=\frac{mass\;of\;dried\;pectin}{mass\;of\;dried\;lemon\;powder}\times 100$$

### 3.3. Pectin Characterization

#### 3.3.1. Degree of Esterification of Pectin (Including Commercial Pectin)

The degree of esterification (DE) of pectin was evaluated as follows [53]. First, 0.1 g of dried pectin was dissolved in 20 mL of distilled water at 40 °C after moistening with 2 mL of ethanol. The solution was titrated with 0.1 M NaOH, using phenolphthalein as a color indicator (V1). Thereafter, 10 mL of 0.5 M NaOH was added, and the mixture was shaken and allowed to stand for 20 min. Then, 10 mL of 0.5 M HCl was added and titrated against 0.1 M NaOH to a faint pink color which persisted after shaking (V2). The DE was calculated using the equation below:

$$\mathrm{Degree}\;\mathrm{of}\;\mathrm{esterification}\;(\%)=\frac{V2}{V2+V1}\times 100$$

#### 3.3.2. Equivalent Weight of Pectin (Including Commercial Pectin)

The equivalent weight of pectin was calculated using the Kute et al. method [54]. Briefly, 0.5 g of pectin was placed in a 250 mL conical flask and moistened with 5 mL of ethanol. Subsequently, 1.0 g of NaCl and 100 mL of distilled water were added, followed by the addition of 5 drops of phenol red indicator. The solution was then titrated slowly with 0.1 N NaOH until the color changed to pink, indicating the endpoint:

$$\mathrm{Equivalent}\;\mathrm{weight}\;(g/\mathrm{mol})=\frac{Weight\;of\;pectin\;(g)}{ml\;of\;alkali\times Normality\;of\;alkali}\times 1000$$

#### 3.3.3. Methoxyl Content of Pectin (Including Commercial Pectin)

The methoxyl content (MeO) of the pectin was measured using the method described by Khamsucharit et al. [55]. In summary, 25 mL of 0.25 N NaOH was added to the neutralized solution obtained from the equivalent weight. The solution was thoroughly mixed and allowed to stand for 30 min at room temperature in a sealed flask. Subsequently, 25 mL of 0.25 N HCl was added and titrated until the color turned pink:

$$\mathrm{MeO}\;\mathrm{Content}\;(\%)\;(\mathrm{mol})=\frac{ml\;of\;alkali\times Normality\;of\;alkali\times 31}{Weight\;of\;sample\;\left(g\right)\times 1000}\times 100$$

#### 3.3.4. Anhydrouronic Acid Content of Pectin (Including Commercial Pectin)

The anhydrouronic acid (AUA) content of the pectin was assessed by following the procedure of Kamal et al. [38]. This involved utilizing a specific formula to calculate the AUA content based on the titration volumes obtained during determination of the equivalent weight and methoxyl content:

$$\mathrm{AUA}\;(\%)=\frac{176\times 0.1z\times 100}{w\times 1000}\;+\frac{176\times 0.1y\times 100}{w\times 1000}\;$$

where the molecular unit of AUA (1 unit) is 176 g, z is the milliliters (titer) of sodium hydroxide from the equivalent weight determination, y is the milliliters (titer) of NaOH from the MeO content determination, and w is the weight of the sample.

#### 3.3.5. Moisture Content Determination of Pectin, Including Commercial Pectin

The method of Lai et al. [56] was employed to determine the moisture content of the pectin. This involved weighing 1 g of powdered pectin and placing it on a metal dish. The pectin was then dried in an oven at 100 °C for 4 h, followed by cooling in a desiccator before reweighing. The final weight of the oven-dried pectin was recorded, and the moisture content was calculated using the equation below:

$$\mathrm{Moisture}\;\mathrm{content}\;(\%)=\frac{Powder\;pectin\;after\;oven-dried\;(g)}{powder\;pectin\;before\;oven-dried\;(g)\;}\times 100$$

#### 3.3.6. Ash Content Determination of Pectin (Including Commercial Pectin)

The oven-dried pectin sample (1 g) was incinerated in a furnace at 600 °C for 4 h, and the ash was cooled and stored in a desiccator. The following equation was used to calculate the ash content:

$$\mathrm{Ash}\;\mathrm{content}\;(\%)=\frac{Ashed\;pectin\;(g)}{Oven-dried\;pectin\;(g)\;}\times 100$$

#### 3.3.7. Fourier Transform Infrared Spectroscopy (FTIR) Analysis

A PerkinElmer (UATR Two) spectrometer was used to analyze the FTIR spectra of the pectin, including the commercial pectin. The range of the obtained spectra was 4000–500 cm^−1^, with a resolution of 4 cm^−1^ and 32 performed scans. This range allowed for the identification of various functional groups present in the pectin, providing insights into its molecular structure.

#### 3.3.8. Microstructure Evaluation

The pectin samples were examined for their morphology using scanning electron microscopy, Tokyo, Japan (JEOL JSM-6390LV instrument). The pectin samples, including the commercial pectin, were coated with gold three times at room temperature for better imaging.

#### 3.3.9. Thermal Properties

The analysis of pectin and commercial pectin using thermogravimetric analysis (TGA) was conducted and documented with a PerkinElmer Pyris 6 TGA instrument (PerkinElmer, Inc., Waltham, MA, USA). The temperature range during the analysis was from 25 °C to 700 °C in a nitrogen atmosphere, with a heating rate of 10 °C per minute.

### 3.4. Evaluation of Pectin Antioxidant Activity Using the DPPH Test

The 2.2-diphenyl-1-picrylhydrazyl (DPPH) radical scavenging activity used in this protocol was described by Assefa et al. [57]. However, with some modifications, various concentrations of pectin (including the commercial pectin) ranging from 0 to 2500 mg/L were prepared, and 2 mL of each pectin solution was mixed with 2.5 mL of DPPH (2 mM) radical solution. A negative control was prepared using a DPPH solution mixed with 50% ethanol instead of the extract. The mixture was vigorously shaken and incubated in the dark for 1 h, as determined from preliminary experiments. The ascorbic acid (standard vitamin C) was used as a positive control. Absorbance readings were taken at 517 nm using a PerkinElmer universal absorption spectrophotometer to calculate the DPPH radical scavenging activity of the pectin and ascorbic acid. The results for the DPPH assay were expressed as IC_50_:

$$\mathrm{DPPH}\;\mathrm{Scavenging}\;(\%)=\frac{Absorbance\;of\;control-Absorbance\;of\;sample}{Absorbance\;of\;control}\times 100$$

where ethanol is a negative control (50% ethanol).

### 3.5. Statistical Analysis

All experiments on pectin (including the commercial pectin) were performed in triplicate. Statistical analysis was carried out using IBM SPSS (version 29.0). Analysis of variance (ANOVA) was used to determine the statistical difference between the samples. The difference was considered statically significant at *p* < 0.05. The results were expressed as the mean ± standard deviation and compared using Duncan’s multiple range test.

## 4. Conclusions

The analysis of pectin which was extracted from two geographic regions in two different years (2023 and 2024) was carried out through various methods such as thermal analysis, physicochemical characterization, and FTIR spectroscopy. The antioxidant studies of the extracted pectin, including commercial pectin, were evaluated using a DPPH assay. The thermal analysis highlighted the pectin’s thermal stability and degradation behavior, indicating its suitability for different processing conditions. The physicochemical properties highlighted the EW, ash content, moisture content, and AUA content determination and its potential functionality in food and pharmaceutical applications. The research on antioxidants showed that pectin has scavenging abilities, indicating its potential as a natural antioxidant with health advantages. This study enhanced our understanding of lemon pectin and its potential uses across different sectors. Future research could delve into further characteristics, interactions with other substances, and its effectiveness in various formulations to improve functionality. By utilizing natural sources like lemon pectin, sustainable and functional ingredients can be developed for a range of industries. Thus, this study revealed valuable insights into the extracted pectin and its potential uses.

## Figures and Tables

**Table 1:** Yield of pectin samples.

Pectin Samples (from Two Lemon Farms and Their Years)	Abbreviation	Maximum Pectin Yield (%)
Fort Beaufort pectin sample 2024	FBP 2024	12.2 ± 0.02 ^a^
Peddie pectin sample 2024	PP 2024	13.0 ± 0.02 ^b^
Peddie pectin sample 2023	PP 2023	13.1 ± 0.03 ^b^
Fort Beaufort pectin sample 2023	FBP 2023	12.2 ± 0.03 ^a^

Data expressed as mean ± standard deviation. Different letters (a and b) in the same column indicate significant differences among samples (*p* < 0.05), where *n* = 3.

**Table 2:** Physicochemical properties of extracted pectin vs. commercial pectin.

Pectin Samples	DE (%)	EW (g/mol)	MeO Content (%)	AUA (%)	Ash Content (%)	Moisture Content (%)
FBP 2024 (Fort Beaufort pectin sample in 2024)	80.6 ± 0.24 ^a^	862,1 ± 6.00 ^c^	19.2 ± 0.02 ^a^	94.2 ± 0.03 ^d^	1.0 ± 0.02 ^a^	10.8 ± 0.03 ^c^
PP 2024 (Peddie pectin sample 2024)	82.7 ± 0.09 ^b^	833.3 ± 9.50 ^ab^	9.3 ± 0.01 ^b^	91.5 ± 0.07 ^c^	1.1 ± 0.01 ^b^	11.1 ± 0.02 ^d^
PP 2023 (Peddie pectin sample 2023)	87.0 ± 0.02 ^c^	841.8 ± 6.51 ^b^	11.8 ± 0.02 ^d^	88.7 ± 0.02 ^b^	1.1 ± 0.01 ^b^	9.6 ± 0.02 ^b^
FBP 2023 (Fort Beaufort pectin sample in 2023)	89.5 ± 0.02 ^d^	983.3 ± 2.93 ^d^	10.9 ± 0.03 ^c^	82.4 ± 0.03 ^a^	1.0 ± 0.01 ^a^	9.3 ± 0.04 ^a^
Commercial P (commercial pectin)	95.5 ± 0.01 ^e^	824.2 ± 6.00 ^a^	13.7 ± 0.02 ^e^	98.9 ± 0.01 ^e^	3.6 ± 0.02 ^c^	9.3 ± 0.01 ^a^

Data are expressed as mean ± standard deviation. Different letters (a to e) in the same column indicate significant differences among samples (*p* < 0.05), where *n* = 3.

**Table 3:** Pectin step degradation with corresponding temperatures.

Pectin Samples	First Step Degradation		Second Step Degradation		Third Step Degradation	
	Temperature (°C)	Weight Loss (%)	Temperature (°C)	Weight Loss (%)	Temperature (°C)	Weight Loss (%)
PP 2024	81.00 °C	9.62%	261.05 °C	36.09%	350.56 °C	16.97%
PP 2023	91.04 °C	12.31%	259.15 °C	32.15%	394.11 °C	17.87%
FBP 2023	86.95 °C	10.18%	263.09 °C	33.66%	345.22 °C	21.91%
FBP 2024	86.43 °C	11.78%	260.45 °C	28.69%	350.55 °C	21.98%

**Table 4:** IC_50_ of all pectin samples vs. ascorbic acid.

Samples	DPPH IC_50_ Values of Pectin Samples (mg/L)	DPPH IC_50_ Value of Ascorbic Acid (mg/L)
PP 2024	1062.5 ± 20.0 ^b^	15.2 ± 0.3
PP 2023	1201.3 ± 22.0 ^c^	
FBP 2023	1304.6 ± 19.0 ^d^	
FBP 2024	1382.6 ± 29.9 ^e^	
Commercial P	1019.4 ± 17.1 ^a^	

Data are expressed as mean ± standard deviation. Different letters (a–e) in the same column indicate significant differences among samples (*p* < 0.05), where *n* = 3.

## Data Availability

The original contributions presented in the study are included in the article, further inquiries can be directed to the corresponding author/s.
